# Modeling vehicle ownership with machine learning techniques in the Greater Tamale Area, Ghana

**DOI:** 10.1371/journal.pone.0246044

**Published:** 2021-02-16

**Authors:** Mohammed Abdul Muhsin Zambang, Haobin Jiang, Lukuman Wahab

**Affiliations:** 1 School of Automotive and Traffic Engineering, Jiangsu University, Zhenjiang, China; 2 Civil Engineering Department, Tamale Technical University, Tamale, Ghana; University of Rochester, UNITED STATES

## Abstract

Vehicle ownership modeling and prediction is a crucial task in the transportation planning processes which, traditionally, uses statistical models in the modeling process. However, with the advancement in computing power of computers and Artificial Intelligence, Machine Learning (ML) algorithms are becoming an alternative or a complement to the statistical models in modeling the transportation planning processes. Although the application of ML algorithms to the transportation planning processes—like mode choice, and traffic forecasting and demand modeling—have received much attention in research and abound in literature, scanty attention is paid to its application to vehicle ownership modeling especially in the context of small to medium cities in developing countries. Therefore, this study attempts to fill this gap by modeling vehicle ownership in the Greater Tamale Area (GTA), a typically small to medium city in Ghana. Using a cross sectional survey of formal sectors workers, data was collected between June–August 2018. The study applied nine different ML classification algorithms to the dataset using 10-fold cross-validation technique/s and the Cohen-Kappa static/statistic to evaluate the predictive performance of each of the algorithms, and the Permutation Feature Importance to examine the features that contribute significantly to the prediction of vehicle ownership in GTA. The results showed that Linear Support Vector Classification (LinearSVC) classifier performed well in comparison with the other classifiers with regards to the overall predictive ability of the classifiers. In terms of class predictions, K- Nearest Neighbors (KNN) classifier performs well for no-vehicle class whiles Linear Support Vector Classification (LinearSVC) and GaussianNB classifiers performs well for motorcycle ownership. LinearSVC and Logistic Regression classifiers performed well on the car ownership class. Also, the results indicated that travel mode choice, average monthly income, average travel distance to workplace, average monthly expenditure on transport, duration of travel to workplace, occupational rank, age, household size and marital status were significant in predicting vehicle ownership for most of the classifiers. These findings could help policies makers carve out strategies that would reduce vehicle ownership but improve personal mobility.

## 1.0 Introduction

Individual mobility is an essential part of any transportation system, which is significantly influenced by the availability of public transport and private vehicle ownership and usage. Furthermore, controlling the rise in private vehicle ownership, and increasing quality and access to public transportation is an indispensable part of any sustainable transportation planning strategy [1, 2]. However, this strategy seems to elude most developing countries in Africa especially Ghana where the public transport is characterized by low-quality services culminating in rising private vehicle ownership with its antecedent problems of heavy congestions on the roads, increase fuel consumption, rise in the number of accidents, increase air and noise pollution. As a result of these adverse effects of private vehicle ownership, several studies have been conducted to model the factors affecting vehicle ownership and usage. These studies mainly use statistical methods which are group into aggregate and disaggregate models. The aggregate models are estimated using zonal level factors to determine the general levels of private vehicle ownership whiles the disaggregate models uses individual or household characteristics to estimate vehicle ownership at the individual or household levels [3, 4]. Typically, these models are used for inferring the factors that influence the level of vehicle ownership pattern and usage. However, the drawback of these statistical models is that a predefined relationship is required between the dependent and the independent variable. In addition, outliers in the dataset, multicollinearity among independent variables and missing values negatively affect the performance of the statistical models. Additionally, these models have low accuracy for the prediction of vehicle ownership to examine future possible policy alternative [5].

As a result of these drawbacks and coupled with the advancement in Artificial Intelligence (AI), Machine Learning (ML) algorithms are becoming an alternative or a complement to the statistical models. In contrast to the statistical models, ML models do not require a predefined relationship with the dependent and independent variables; instead, it learns complex relations between the dependent and independent variables by automatically developing a mapping function given an input data. In other words, ML algorithms learn a target function that best maps the input variables to the outcome variables without necessary predefining the mathematical structure. The advantage of the ML algorithms lies in its ability to detect the general trend and patterns within a given data to predict the target variable. It also has the ability to deal with missing data, outliers and multicollinearity among the feature variables [6–9]. Although the ML algorithms produce accurate predictions over that of the statistical models, it does not provide a reasonable theoretical interpretation of the model results.

Despite the inability of some of the ML algorithms to provide inference for the model results, its usefulness has been demonstrated in many fields of research including the transportation domain as a complement or replacement/alternative to existing statistical models. For instance, at the city-scale transportation level, ML algorithms have been applied in estimating traffic speeds [10–13], traffic volume estimation [14–16], demand prediction such as city level bike sharing demand prediction [17, 18] and traffic reconstruction and visualization [19] are examples among other subdisciplines in the transportation domain.

In terms of mode choice modeling, Hensher and Ton [20] explored the predictive ability of Artificial Neural Network (ANN) and Nested Logit Model (NLM) in modelling commuter mode choice. Their results did not show any significant difference between the ANN and the NLM. Their findings could have been affected by the limited/ utilization of the property of distributed memory of ANN. However, other studies (see [21–25]) found that ANN outperforms the conventional statistical methods like Multinomial Logit (MNL) and NLM to modeling travel mode choice.

Apart from ANN, other ML algorithms have been successfully applied to mode choice modeling. Zhang and Xie [26] demonstrated, for the first time, the use of Support Vector Machine (SVM) to travel mode choice modeling using data collected in the San Francisco Bay Area in California and compared it to ANN and MNL. Their results show that SVM outperforms ANN and MNL, and they proposed that SVM should be adopted over ANN as it is easier and straightforward to implement. Later studies, however, by Omrani [27] who investigated the predictive performance of ANN, SVM and MNL shows that ANN outperforms all the other algorithms. Hagenauer and Helbich [28] also compared the predictive performance of seven ML algorithms for mode choice analysis and found that Random Forest (RF) outperforms both ANN and SVM, including the traditional method of MNL.

Although the modeling framework of both mode choice and vehicle ownership using the traditional statistical approach, especially when using disaggregate data, are similar as both commonly use the theory of utility maximization for the formulation of the mathematical structure for parameter estimation [29, 30], the application of vehicle ownership modeling to ML is limited as shown by Karlaftis and Vlahogiami [5] in their comprehensive review of statistical and ANN modeling approach to transportation research with only one study by Mohammadian and Miller [31] that compared the predictive power of NLM and ANN. Their findings indicated that both models generated a strong result with the ANN having better predictive potential than the NLM. A further search on literature on the application of ML to vehicle ownership modeling found only a few other papers that have used ML as the time of writing this article. For instance, Paredes et al. [32] compared five ML algorithms (i.e. RF, SVM, Decision Tree(DT), Extreme Gradient Boosting(EGT) and an Ensemble of Methods) to MNL in terms of vehicle ownership estimation and prediction using household survey data from Singapore. Their results show that ML algorithms outperform the MNL model with RF having the highest performance in terms of accuracy, noting that ML and MNL are not interchangeable but complementary in terms of vehicle ownership modeling. Ha et al. [33] also examined the feature impact level of ANN, RF and MNL to predict vehicle ownership levels in the city of Phnom Penh. Their results indicated that household income was the most prominent feature affecting vehicle ownership in Phnom Penh and that the RF produced the highest accuracy in terms of prediction. Kaewwichiann et al. [34] also explore the use of DT and ANN for vehicle ownership modeling using household survey data from Khon Kaen Province, Thailand. Their results indicated that ANN outperforms DT.

The above review of modeling vehicle ownership with ML techniques indicates that only limited studies have applied ML. Also, these studies have used a limited set of ML algorithms, although the number of available algorithms are many. Additionally, these studies produced different results in terms of the best performing algorithms, making the application of ML algorithms case-specific. Therefore, this study seeks to expand on the literature of using ML techniques to model vehicle ownership especially in the context of small to medium city in a developing country like Ghana where disaggregate data on individual vehicle ownership are usually inadequate for disaggregate vehicle models. Consequently, this study aims to evaluate the use of machine learning techniques in modeling and predicting vehicle ownership levels in Ghana using Greater Tamale Area (GTA) as the case study and to identify the factors that are significantly influencing vehicle ownership levels. The results of this study will provide critical information to policymakers as to the most influencing factors affecting vehicle ownership levels and to carve out policies that would help reduce vehicle ownership but improve personal mobility. Additionally, it will add on to the limited literature on the application of ML to vehicle ownership especially in the context of a small to medium city in a developing country.

The rest of the article is structured as follows. In addition to the introduction presented in section one, we present a brief description of the study area and the transportation system in section two. This is followed by a discussion on the dataset and modelling framework in section three. In section four, the model estimation results and discussions are presented whiles section five present the conclusions and recommendations.

## 2.0 Study area and transport system

The geopolitical boundaries of the Tamale Metropolitan Assembly (TaMA) and the Sagnarigu Municipal Assembly, which was carved out of TaMA in 2012 for easy administration and deepening of local government as a result of remarkable expansion of the city of Tamale over the years, is what is referred to as the Greater Tamale Area in this paper. It has an estimated population of about 468,318 as at the end of 2018 [35] and a land size of about 922km^2^. The city is centrally located within Northern Ghana and doubles as the capital city of the Northern Region. It is also the third largest city in Ghana after Accra and Kumasi. As a result, GTA host many government offices, private companies, research, and educational institutions.

GTA also has a unique transportation system and infrastructure that is entirely different from the southern part of Ghana. Shared-Taxis and Shared-Tricycle-Taxis dominate the public transportation system for intracity transport. The mini-buses known as ‘trotro’ that is usually used in the country’s big cities for intracity commuting purposes rather provide intercity transport services in GTA. As a result, commuters are left with the choice of either using this form of public transport (i.e. Shared-Taxi or Shared-Tricycle Taxis) or using personal means of transport for their daily commuting needs.

Additionally, the road network in GTA is such that all the principal streets and some linking roads in the city is/are provided with segregated bicycle and pedestrian lanes with most linking roads also having side lanes for both pedestrians and cyclists; making GTA a non-motorized transport friendly city. However, the share of the non-motorized means in the city is switching to motorized means like motorcycles and cars in contrast to the situation in the early 1990s and 2000 where cycling was the most common mode of transport in GTA with its use not only limited to the poor but as well as the well to do [36]. Consequently, there is an ascendency in the use of private vehicles for the daily commuting needs of the residents. This has resulted in increasing levels of congestion and traffic jump in the metropolis culminating in the government proposing to build an interchange within the Central Business District (CBD) of GTA [37]. Therefore, studying the factors that affect vehicle ownership would help city-planners in fashioning policies that will be aimed at reducing the rising numbers of the use of personal vehicles and to develop strategies that will take advantage of the non-motorized infrastructure to encourage commuters to switch to the non-motorized mode of transport.

## 3.0 Dataset and modeling framework

### 3.1 Dataset

The dataset for this study was gotten from a structured revealed preference survey conducted on a cross-section of formal sector workers within GTA from June to August, 2018, using KoBo Toolbox an online open-source suite of tools for field data collection developed by the Harvard Humanitarian Initiative, Brigham and Women’s Hospital and Kweyo with support from other organizations. This group of workers were selected because they have a stable monthly income making them easy targets for banks/financial institutions or vehicle dealers in the country to offer them vehicle loans or high purchase agreements for vehicles, thereby, contribute significantly to the overall private vehicle ownership within the country.

The survey was conducted on both public and private institutions. The Public institutions included Government Departments and Agencies (GDA) that included the Department of Urban Roads, Metropolitan Assembly, Survey Department, Land Commission, Ghana Water Company, Tamale Technical University (TaTu), University for Development Studies-Tamale Campus (UDS), Tamale Central Hospital (TCH), Tamale Teaching Hospital (TTH) and Ghana Education Service (GES). The private institutions included Tecno Mobile, Vodafone Ghana, and Ghana Developing Communities Association (GDCA). These institutions were selected for the study because they employed a substantial number of Formal workers in GTA and located on different parts of the city. Majority of their staff also depend on either private or public means of transport to get to work.

The survey solicited information on socio-economic characteristics of respondents like age, household size, gender, job rank, level of education, monthly income as well as travel characteristics like level of vehicle ownership, average travel time to work, average travel distance to work, choice of mode to work, reasons for choosing a particular mode and average monthly expenditure on transport. In terms of transport infrastructure, respondents were asked as to whether the road network had enough provision for non-motorized transport and whether they would be willing to switch to either walking or cycling. Also, respondent’s willingness to switch from their current mode of choice to work to a Metro Bus Service if it was available and the reasons for willingness to switch or not were also solicited. For spatial and population density, respondents living within 2km radius from the CBD were taken as living in a dense area.

The survey was conducted directly at the selected public and private institutions using three android tablets. Before entry into each institution, permission was sought from institutional and departmental heads. For each institution or department, an introductory letter was given to the researcher by the heads explaining, among other things, the aims, and procedures of the research to prospective participants. In addition, a written consent, implied by agreeing to complete the study questionnaire, was obtained from each participant. No intervention or treatment was administered to any participant, and there was no manipulation of any participant or study environment. All participants were assured of anonymity and privacy and informed of their rights to withdraw from the study anytime without any adverse consequences. A secure online data collection tool (KoBo Toolbox), also available offline, was used for data collection. The data was downloaded later unto a password-protected computer accessible only to the research team members. The procedures of the study were approved by the Ethics and Research Review Committee of the Tamale Technical University. In all, a total of 441 respondents were used.

Three levels of classification for vehicle ownership were identified; “does not own a vehicle”, “motorcycle” and “car”, for the reason that “more than one motorcycle”, “more than one car”, and “both car and motorcycle” ownerships were less than ten respondents. As a result, the car ownership class exclusively included the few respondents who had both cars and motorcycles whiles the motorcycle ownership also exclusively included respondents who had more than one motorcycle. Also, the motorcycle class included all types of motorcycles like mopeds, scooters and motorbikes whiles the car class is all types of vehicles used as a personal means of transport.

Based on the survey data as shown in Table 1, more than half of the respondents (53.6%) owned a motorcycle whiles about 22.2% and 24.0% of the respondents had a car and no-vehicle ownership respectively. Majority of the respondents also depended on a motorcycle or a car for their daily commuting needs accounting for about 51% and 23% respectively. About 9% of the respondents either used non-motorized or tricycle-taxi with about 8% using shared taxi. Credence is given to the fact that lack of efficient public transportation system promotes the use of private vehicles as about 48% of the respondents were willing to switch from their current mode of transport to a Metro Bus Service if it was available. In other words, had there been an efficient public transportation system in the city, the use of private vehicles might have been minimal.

**Table 1:** Output and feature variables.

Variable	Definition	Mean (Std. Dev)
	**Output variable**	
No vehicle	1 if the respondent owns no vehicle otherwise 0	0.240
Motorcycle	1 if respondent owns motorcycle otherwise 0	0.536
Car	1 if the respondent owns a car otherwise 0	0.222
	**Feature variables**	
Income	Average monthly income of the respondent in GH¢1,000.00	1.58 (1.04)
Time	Average travel time to work in minutes	20.77 (12.81)
Expenditure on transport (METT)	Average monthly expenditure on transport in GH¢1,000.00	0.18 (0.20)
Distance to work	Average travel distance to work in kilometres	5.97 (4.18)
Mode Choice	1 if respondents mode choice is Walking/Bicycle otherwise 0	0.09 (0.01)
	1 if respondents mode choice is motorcycle otherwise 0	0.51 (0.02)
	1 if respondents mode choice is car otherwise 0	0.23 (0.02)
	1 if respondents mode choice is tricycle-taxi otherwise 0	0.09 (0.01)
	1 if respondents mode choice is taxi otherwise 0	0.08 (0.01)
Non-motorized infrastructure (BLanes)	1 if the respondent agrees that there is adequate provision of non-motorized infrastructure, otherwise 0	1.33 (0.47)
Density	1 if the respondent is living 2km from the Central Business District, otherwise 0	0.40 (0.49)
Bus Service (MBus)	1 if the respondent is willing to shift to a Metro Bus service otherwise 0	0.48 (0.50)
Gender	1 if respondent is a male, otherwise 0	0.62 (0.49)
Age	Age of the respondents (years)	35.29 (8.05)
Marital Status	1 if married, otherwise 0	0.69 (0.46)
Household Size	Number of people residing in the respondent’s household	4.29 (2.68)
Number of Children (CHS)	Number of children in the respondent’s household	1.93 (1.72)
Occupational Rank (OcR)	1 if senior staff, otherwise 0	0.61 (0.49)

On average, respondents travelled about 6km to get to their work destinations with an average travel time of 20 minutes. Also, about 60% of the respondents lived more than 2km from the CBD and spent about GHȼ180.00 (equivalent to $37.5) out of an average monthly income of ȼ1580.00 ($392.17) on transport. In terms of demographic characteristics, majority of the respondents were males (69%) and married (62%), with an average family size of 4.2. Additionally, the majority of the respondents were between the ages of 20–40, an indication that the sample is more youthful and represented by age as defined by Ghana Statistical Service (GSS) [38].

### 3.2 Modeling framework

Scikit-Learn [39], an efficient and simple tool for data analysis and ML algorithms in Python was used to analyze the dataset. This tool was chosen because it is accessible to everyone and can be applied to several scenarios with a lot of flexibility. It is also an open-source Python library with a well-documented API for easy implementation of ML algorithms [40]. The experiments were carried out using Python 3.7.3 Jupyter Notebook on an ASUS FL5900U laptop with Intel processor i76500U quad core of 2.5GHz, 20 GB RAM, 64-bit Windows 10 pro operating system and an Intel HD Graphics 520 with NVIDIA GeForce 940MX.

Supervised classification ML algorithms were used in this experiment for the reason that the output or target (i.e. vehicle ownership level) variable in our dataset is a labeled categorical variable with three possible outcomes (no-vehicle-ownership, motorcycle and car). Details of the dataset and the feature descriptions are shown in Table 1. The various classifiers were selected based on previous applications to transportation research [41] and for the reason that the structure and shape of different ML algorithms differ in the assumptions of the best methods used to optimize a representation of the learning process. Therefore, there is the need to apply different ML algorithms to the dataset to determine the best approach to estimating the structure of the underlying function that best approximate to the dataset [42]. The following is a brief explanation and the specification for algorithms used in this study.

#### 3.2.1 Logistic regression (LR)

Logistic regression which is known as the maximum-entropy classification, is a type of linear model used for the classification of dependent categorical variables based on one or more independent features. It can handle both binary and multiclass outcomes with options for regularization which penalizes the model in order to prevent overfitting [43]. The ‘newton-cg’ solver is specified in this study which handles l_2_ regularization and converges faster for high dimensional data. The probabilities describing the outcome of a single trial is based on the logistic regression function. However, it must be noted that this does not follow the behavioral specification or the Random Utility Models specifications in the statistical approach. Instead, features are taken uniformly for all target outcomes as a single weight trained for each feature for each target class [41].

#### 3.2.2 Stochastic gradient decent classifier

The stochastic gradient decent algorithm is another linear model that is used to classify dependent categorical outcomes using a regularized linear model with the gradient loss calculated on each sample to reduce the decreasing strength schedule (learning rate). It can be estimated on either dense or sparse data sets and can allow mini-batch learning [44]. It supports multi-categorical classification using the ‘one-verses-all’ method. The model fit is controlled by the loss pentameter. In this study, the ‘modified-huber’ loss is specified which is a smooth loss that allows for both tolerance to outliers and probability estimates [40]. The default value for the learning rate is maintained as this gives the best result in Scikit-Learn.

#### 3.2.3 Support Vector Machine (SVM)

SVM is one of the most popular and versatile machine learning algorithms that is used for supervised learning for both classification and regression tasks. It can analyze both linear and non-linear relationships between the target variable and the independent features by using the principle of structural risk minimization [26]. It constructs a hyperplane in a high-dimensional space which results in an optimum separation between different targets variables. It uses the kernel trick to determine linear or nonlinear hyperplane depending on the nature of the input data. The kernel function can be specified as linear, sigmoid, polynomial and radial basis functions. However, SVM is prone to errors for unscaled data [45]. For this study, the LinearSVC algorithm in Scikit-Learn is used to model the dataset as it supports both dense and sparse input. It handles multiclass using one-vs-the-rest structure. Additionally, the data was scaled using the standard feature Scaler function in Scikit-Learn before the experiments.

#### 3.2.4 Decision tree (DT)

Like SVMs, Decision Trees are also multipurpose non-parametric supervised learning algorithms that can implement classification and regression tasks, and even multi-output outcomes. It uses simple decision rule to learn from the data features that can predict the outcome of the target variable. The merits of this algorithm lie in its simplicity and the ability to produce interpretable models. However, it is prone to overfitting the data thereby reducing the generalizability of the model and can also be unstable if there is a small variation to the data [45]. Several algorithms are used to model the learning process in DT; Scikit-Learn however uses the CART (Classification and Regression Trees) algorithm for the implementation of DT which uses the feature and threshold to construct a binary tree that yields the best information gain at each node. For this study, the entropy information gain criterion for building the tree is used and a maximum depth of three trees is specified in order to reduce the risk of overfitting the model [39].

#### 3.2.5 Ensemble methods

The ensemble learning methods is one of the popular learning algorithms that are applied in many disciplines to make predictions of data. It aggregates the predictions of many base estimators to produce a single estimator that is more robust and a better generalization of the data. It is categorized into two methods, averaging methods which average the predictions of the base estimators to reduce the variance and boosting methods which try to remove the bias of the combined estimator [42]. For this study, two averaging methods based on randomized decision trees; the Random Forest (RF) algorithm and the Extremely Randomized Trees (ERT) method are used whiles Adaboost a popular method for boosting developed by Freund and Schapire [46] is used. The RF ensemble is built using 15 estimators whiles the ERT ensemble is built using five estimators. The Adaboost ensemble is built using the default base estimator which is DT with a maximum depth of three trees.

#### 3.2.6 Naive Bayes (NB)

The NB supervised learning classification algorithms are based on the Bayes theorem which state that the likelihood of a target outcome corresponding to a group of features is conditionally independent of each other; thus, considering all features to contribute independently to the probability of the target outcome. Therefore, the model predicts its probability condition on several independent features. The parameters in the algorithm are estimated using the maximum likelihood method. The different NB classifiers differ mainly due to the assumption regarding the probability distribution function [47]. For this study, the GaussianNB is used which assumes the likelihood of the features to be Gaussian.

#### 3.2.7 K- Nearest neighbors (KNN)

The KNN is an instance-base-learning algorithm that stores the instances of the training data by computing each point using majority vote of the nearest neighbors and assigned the target class with the most representative nearest neighbors. It does not actually construct a model to make predictions. Instead, predictions are made base on the largest proportion of k-nearest point, as a result it is often called a lazy classifier [43]. The study used the KNeighborsClassifier in Scikit-Learn with the default number of five neighbors as this produced the best accuracy results. Additionally, the features were scaled before fitting the model as distance calculations between the neighbors are considered on the same scale.

### 3.3 Feature importance and ranking evaluation

As was pointed out in the introduction, one major challenge of ML algorithms is the inability of some of the algorithms to produce interpretable results. However, other algorithms have some attributes that can be used as a form of interpretation for the model results. For instance, algorithms with the ability to produce feature importance or feature ranking relative to the target variables can form the bases for some form of interpretations. The feature importance or ranking is the amount of contribution an input variable has on the overall predictive ability of the ML model relative to the outcome. For this study, the Permutation feature importance is used to evaluate the feature importance for each of the classifiers. This method was chosen because it can handle biases from categorical variables with many categories preferred. Permutation feature importance is the reduction in model score when the value of a single feature is arbitrarily shuffled. In other words, it is the measure of how scores of an estimator reduce when a feature is excluded from the model estimation. A reduction in the model scores indicates how important this feature is, in the overall performance of the model (see [23, 34, 48] for more details).

### 3.4 Feature transformation

As shown in Table 1, the dataset is made of both continuous and categorical features. However, most ML algorithms are unable to handle categorical features, therefore, it is necessary to convert all categorical features to numeric values before inputting them into the ML algorithms. An efficient way of converting the categorical features into numeric values is the use of One Hot Encoding which splits the categories into as many columns as the number of categories present in the original feature and then assigns a zero to all the rows that did not have a particular category and one for the row that did have that category. The advantage of this approach is that, it does not result in a situation where the ML algorithms will interpret the categories as ordinal [43]. However, this approach increases the dimensionality of the dataset which might generate overfitting results, a situation known as “the curse of dimensionality”. To tackle this challenge in this experiment, the Linear discriminant Analysis (LDA) was used to transform the features before inputting the features into the selected algorithms for model selection and evaluation.

### 3.5 Models evaluation and performance metric

According to Kohavi [49], 10-fold stratified cross-validation is the best method for model selection and evaluation. This method divides the data set into ten random folds whiles preserving the distribution of the data by maintaining the class percentages of the samples. The model is then trained on ninth-tenth of the fold and the result is then validated using the remaining fold to calculate the model performance and this process repeated nine times for each of the other folds. The superiority of this technique, is that it accounts for any bias that could be as a result of splitting the data into only training and test set [43]. Therefore, this study adopted the 10-fold stratified cross-validation approach to evaluate the model performance for each of the classification algorithms used. The data set was divided into a training set of 70% and testing set of 30% before applying the 10-fold stratified cross-validation approach for the evaluation of the selected algorithms.

The performance metrics used from the confusion matrix for each of the classifiers are the area under the curve for the Receiver Operating Characteristic curve (ROC-AUC), recall, accuracy, precision, sensitivity, and specificity. A typical sample of a confusion matrix is shown in Table 2 with the True Positive (TP) representing the number of positive responses that were correctly classified as positive response whiles False Positive (FP) is the number of negative responses that were wrongly classified as a positive response. On the other hand, True negative is the number of negative responses that were correctly classified as negative response whiles False Negatives (FN) is the number of positive responses that was wrongly classified as a negative response. Sensitivity is the per cent of positive response that were correctly classified whereas specificity is the per cent of negative response that were correctly classified. Accuracy measures the overall correctness of the model whereas precision measures the proportion of correctly classified positive response. Recall, which is the same as sensitivity in its calculation, measures the completeness of the model with higher recall values indicating fewer FN [43]. The ROC is the plot of sensitivity (True Positive Rate) against 1-specificity (False Positive Rate) which summarizes the performance of the classifier over all possible threshold with ROC-AUC closer to 1 indicating an excellent performance over many thresholds [50]. Additionally, another performance measure used to evaluate the performance of the classifiers is the Cohen-Kappa static which is an agreement index measure between the prediction and the actual class with values ranging from -1 to 1. Values closer to 1 indicate higher model performance [51]. The formula for calculating each of the metrics are given below.

$$Precision=\frac{TP}{TP+FP}$$ (1)

$$Sensitivity=Recall=\frac{TP}{TP+FN}$$ (2)

$$Specificity=\frac{TN}{TN+PF}$$ (3)

$$Acuracy=\frac{TP+TN}{TP+TN+PF+FN}$$ (4)

$$Cohen-Kappa=\frac{P_{0}-P_{e}}{1-P_{e}}$$ (5)

Where P_o_ is the observed agreement and P_e_ is the expected agreement by chance with the true class, respectively.

**Table 2:** Confusion matrix.

		Predicted	
	Response Type	Positive response	Negative response
Actual	Positive response	True positive (TP)	False Negative (FN)
	Negative response	False positive (FP)	True Negative (TN)

## 4.0 Results and discussion

**Fig 1:**
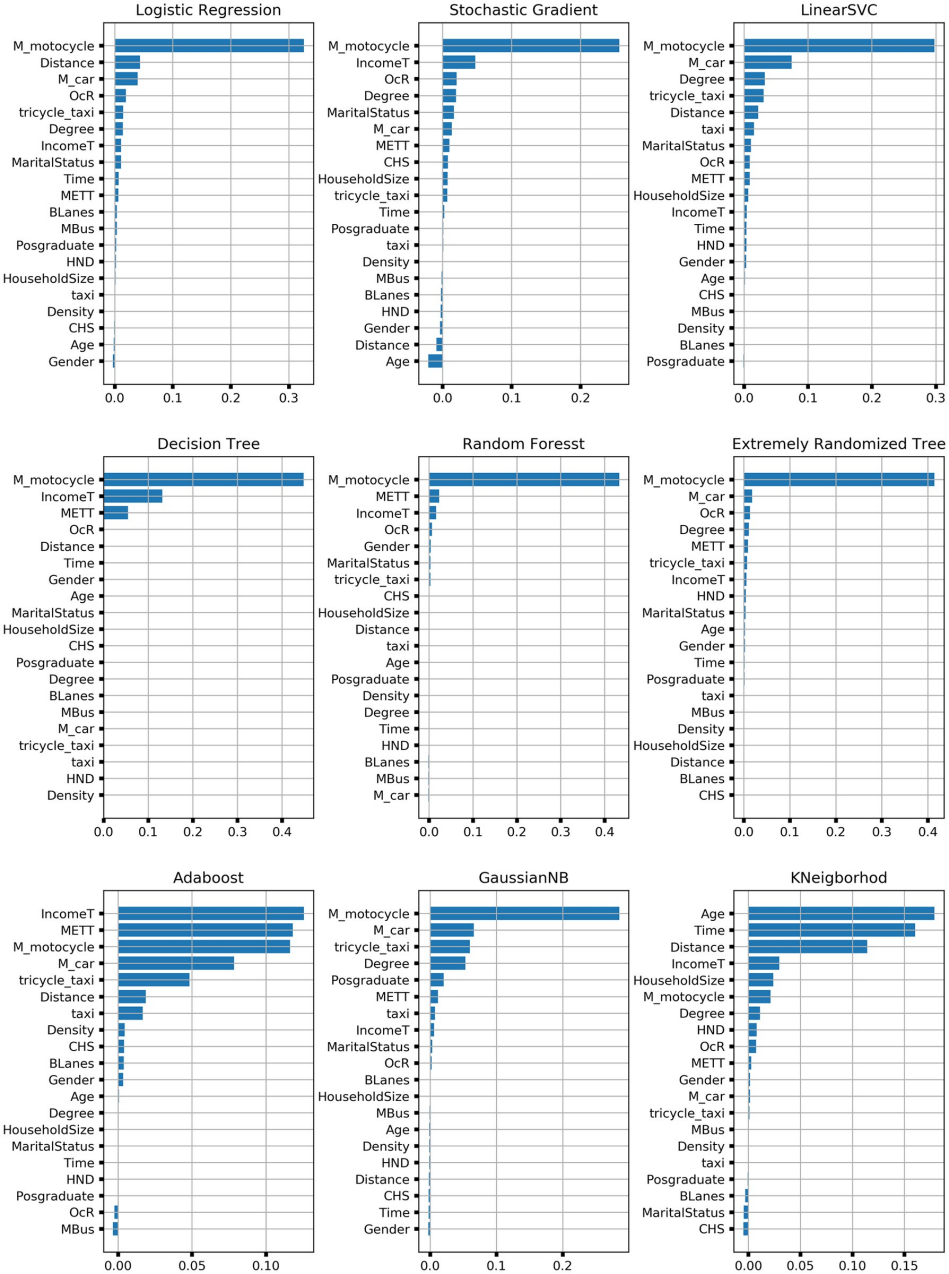
The figure for the permutation feature importance for the various classifiers.

To prone down the number of features so as to reduce overfitting the algorithms to the data set and to assess the level of contribution the features in predicting the vehicle ownership classification in GTA, permutation feature important is apply to the selected algorithms as shown in Fig 1. Respondent’s mode choice of the motorcycle is the most significant feature for many of the classifiers except for Adaboost and KNN classifier with the most significant feature being average monthly income and age of the respondents, respectively. For Stochastic Gradient Decent (SGD), DT and Random Forest (RF) classifiers, the average monthly income is the second most important feature for prediction of vehicle ownership in GTA. DT classifier only used three features namely motorcycle mode choice, income, and monthly expenditure on transport as the most important features. Distance travel to respondent’s workplace is the second most important feature for logistic regression (LR), the third most important feature for SGD and KNN and fifth most important feature for Linear Support Vector Classification (LinearSVC), respectively. Time is only a strong significant feature in the KNN classifier ranking second as the most important feature. Monthly expenditure on transport placed third as the most important feature for DT, RF and Adaboost classifiers, respectively. Apart from the age and household size which placed first and fourth, most important feature for the KNN classifier, most of the socio-demographic characteristics of the respondents were less significant in predicting vehicle ownership in GTA. In all, respondents travel characteristics like mode choice, distance travelled to workplace and duration of travel, average monthly income and occupational rank placed between the first and fifth most important feature for most of the classifiers. Provision of non-motorized transport infrastructure, willingness to switch from current transport mode to work to Metro Bus Service if available and density were less important in predicting vehicle ownership for all the classifiers. Therefore, the features that is included in the final analysis of the algorithms are the top five features from the various classifiers in the feature important ranking for the training and testing of the various classifiers.

Table 3 shows the performance metrics for each of the classifiers. The Linear LinearSVC classifier produced the highest average prediction accuracy of 94%. Closely followed by the LR, Extremely Randomized Trees (ERT), GaussianNB and Adaboast classifiers which produced an average prediction accuracy of 93%. The DT, SGD, KNN and RF classifiers gave an accuracy of 92%. The same result is recorded for average precision and recall for each of the classifiers. However, in terms of class categories, for the no-vehicle class, the highest precision of 97% is achieved by the KNN classifier.

**Table 3:** Confusion matrix and performance metric by class for the classifiers.

Classifier		Confusion Matrix			Pr	Re	Acc	Kappa
	Actual Class	Predicted Class						
Logistic Regression (LR)		No Vehicle	Motorcycle	Car				
	No Vehicle	32	1	1	0.88	0.94	0.93	0.89
	Motorcycle	2	66	2	0.97	0.94		
	Car	0	1	28	0.90	0.90		
Stochastic Gradient (SGD)	No Vehicle	31	1	2	0.91	0.91	0.92	0.88
	Motorcycle	1	67	2	0.96	0.96		
	Car	2	2	25	0.86	0.86		
LinearSVC	No Vehicle	32	1	1	0.91	0.94	0.94	0.90
	Motorcycle	2	66	2	0.97	0.94		
	Car	1	1	27	0.90	0.93		
Decision Tree (DT)	No Vehicle	31	1	2	0.94	0.91	0.92	0.86
	Motorcycle	1	66	3	0.94	0.94		
	Car	1	3	25	0.83	0.86		
Random Forest (RT)	No Vehicle	31	1	2	0.91	0.91	0.92	0.88
	Motorcycle	1	67	2	0.96	0.96		
	Car	2	2	25	0.86	0.86		
Extremely Randomized Trees (ERT)	No Vehicle	31	1	2	0.93	0.92	0.93	0.89
	Motorcycle	1	66	3	0.96	0.94		
	Car	2	1	26	0.88	0.93		
Adaboost	No Vehicle	31	1	2	0.91	0.91	0.93	0.89
	Motorcycle	1	68	1	0.96	0.97		
	Car	2	2	25	0.89	0.86		
GaussianNB	No Vehicle	32	1	1	0.91	0.94	0.93	0.89
	Motorcycle	2	65	3	0.97	0.93		
	Car	1	1	27	0.87	0.93		
K-Neighbors (KNN)	No Vehicle	31	2	1	0.97	0.91	0.92	0.88
	Motorcycle	1	66	3	0.93	0.94		
	Car	0	3	26	0.87	0.90		

Pr = precision, Re = recall and Acc = Accuracy

On the other hand, the highest recall of 94% is achieved by the LR, LinearSVC and GaussianNB classifiers whiles the lowest precision of 88% is attained by LR classifiers, and a lowest recall 91% is attained by the DT, SGD, RT, Adaboost and KNN classifiers. The motorcycle ownership class recorded the highest precision of 97% by the LR, LinearSVC and GaussianNB classifiers, and recall of 97% by the Adaboost classifier whiles the car ownership category achieved the highest precision of 90% for LR and LinearSVC classifiers and recall of 93% for ERT, GaussianNB and LinearSVC classifiers respectively. The lowest precision and recall for the motorcycle ownership class of 93% was achieved by the KNN and GaussianNB classifiers respectively, whiles the car ownership class attained a lowest precision of 83% by the DT classifier, and lowest recall of 86% for the Adaboast, SGD, DT and RT classifiers.

With regards to the Cohen-Kappa static, the highest performing classifiers in terms of prediction agreement to the actual class labels is the LinearSVC with a kappa static of 0.90 whiles the least performing classifier is the DT classifier having a kappa static of 0.86. LR, ERT, Adaboost and GaussianNB produced a kappa value of 0.89 whiles SGD, RT and KNN had a value of 0.88, respectively. Overall, all the classifiers performed well in terms of motorcycle ownership prediction with the least precision and recall of 93% recorded for the KNN and GaussianNB classifiers respectively and performed west for the car ownership prediction with the least precision and recall of 83% and 86% recorded for the DT classifier and the Adaboast, SGD, DT and RT classifiers respectively.

**Fig 2:**
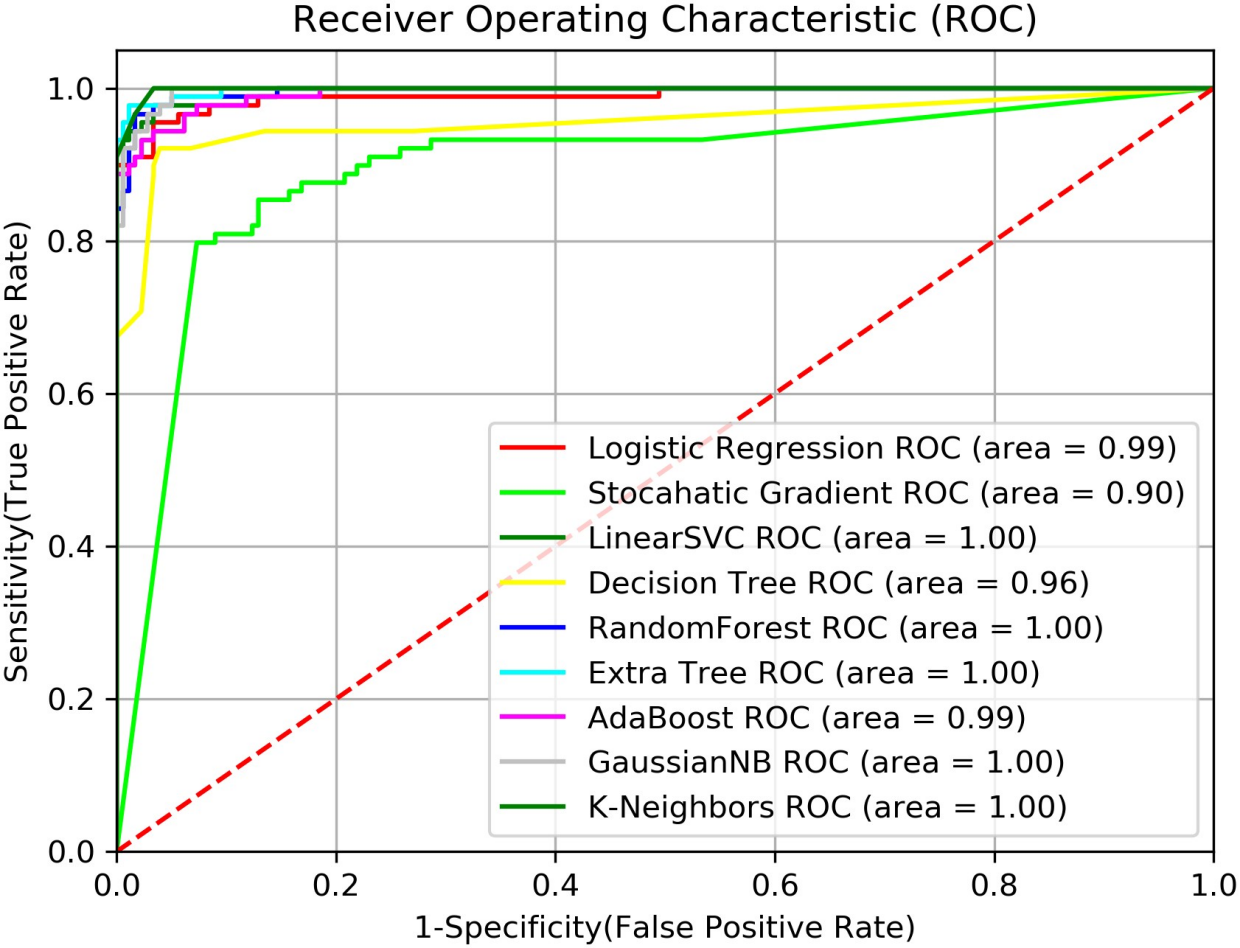
The figure for the Receiver Operating Characteristic (ROC) curves for the various classifiers.

The best performing classifiers over all the possible classification threshold for the ROC curve as shown in Fig 2 are the LinearSVC, ERT, GaussianNB and KNN with an AUC of 1, while the least performing classifier is the SGD. In terms of all the performance metric, the SGD, DT, RT and KNN had the lowest performance in comparison with all other classifiers whiles LinearSVC is the best classifier with an accuracy of 94%, AUC of 1 and a Cohen-Kappa static of 0.90. With regards to class predictions based on precision, KNN performs well for no-vehicle class whiles LinearSVC and GaussianNB performs well for motorcycle ownership class compared to the other classifiers. For car ownership class, LinearSVC and LR performs well in comparison with the other classifiers.

## 5.0 Conclusion

Vehicle ownership modeling and prediction is one crucial task in the transportation planning processes which traditionally uses the statistical modeling techniques in the modeling process. However, with the advancement in computing power of computers and AI, ML algorithms are becoming an alternative or a complement to the statistical models in modeling the transportation planning processes. Although the application of ML algorithms to the transportation planning processes; like mode choice, traffic forecasting and demand modeling have received much attention in research and abound in literature, scanty attention is paid to its application to vehicle ownership modeling especially in the context of a small to medium cities in developing countries.

Therefore, this study attempts to fill this gap by modeling vehicle ownership in GTA, Ghana; using a cross sectional survey of formal sectors workers collected in June–August 2018. The study used LR, SGD, LinearSVC, DT, RF, ERT, Adaboast, GaussianNB and KNN ML classification algorithms to model the dataset. The performance of each classification algorithms was evaluated using accuracy, precision, recall, ROC, and Cohen-Kappa static. Additionally, permutation feature importance was used to determine the features that are significant in predicting vehicle ownership in GTA.

The results show that LinearSVC is the best performing classifier in terms of accuracy of predictions whiles LinearSVC, RF, ERT, GaussianNB and KNN are the best classifiers base on ROC. The best classifier with regards to the Cohen-kappa static is LinearSVC. Therefore, based on accuracy, ROC and Cohen-kappa static, LinearSVC is the best classifier for the dataset. For predictions based on class performance, KNN performs well for no-vehicle class whiles LinearSVC and GaussianNB performs well for motorcycle ownership. For the car ownership category, LinearSVC and LR performs well in comparison with the other classifiers.

The results also indicated that individual mode choice (car or motorcycle), average monthly income, average travel distance to workplace, average monthly expenditure on transport, duration of travel to workplace, age, household size and marital status were significant in predicting vehicle ownership for most of the classifiers. However, provision of non-motorized infrastructure, density, and willingness to switch from current transport mode to work to Metro Bus Service if available were less significant in predicting vehicle ownership for all the classifiers. Our results also demonstrated that ML can also be applied to small to medium cities in developing countries like Ghana.

The findings in this study throws more light on vehicle ownership in the context of a small to medium urban city in Ghana which is mainly influence by the respondents travel characteristics and average monthly income. This finding is largely in agreements with the studies by Ha et al. [33], Kaewwichiann et al. [34] and Paredes et al. [32], however, with regards to the best performing ML algorithm, the finding in our study produced different results which might be as a result of the difference in the data structure and the methods used to find the best learning features for each algorithm, highlighting the case-specific nature of the application of the ML algorithms to vehicle ownership studies.

Based on the most significant features in predicting the vehicle ownership level in GTA, policy makers should look at ways to reduce individual mode choice but, improve the public transportation such as the development of structural public transportation projects such as Bus Rapid Transit, Light Rail or simply a network of regular buses instead of the current informal public transportation (Shared-Taxis and Shared- Tricycle Taxis), which could potential drive down the vehicle ownership level in the city.

## Supporting information

S1 Data The jupyter notebook used for the study which includes the codes for the study in Python 3.7.3. (IPYNB)

S1 File The dataset for the features used for the study. (CSV)

S2 File Features training set. (CSV)

S3 File Features testing set. (CSV)

S4 File The dataset for the outcome used for the study. (CSV)

S5 File Outcome training set. (CSV)

S6 File Outcome testing set. (CSV)

S7 File Description of the feature variable. (CSV)

S8 File The dataset for the outcome used for the study. (CSV)
